# Bis[1,1′-(1,3-phenyl­enedimethyl­ene)di(1*H*-imidazol-3-ium)] β-octa­molybdate

**DOI:** 10.1107/S1600536811053050

**Published:** 2011-12-14

**Authors:** Xiao-Dan Wang, Guang-Feng Hou, Ying-Hui Yu, Jin-Sheng Gao

**Affiliations:** a Engineering Research Center of Pesticides of Heilongjiang University, Heilongjiang University, Harbin 150050, People’s Republic of China, and College of Chemistry and Materials Science, Heilongjiang University, Harbin 150080, People’s Republic of China

## Abstract

In the title compound, (C_14_H_16_N_4_)_2_[Mo_8_O_26_], the β-octa­molybdate anion is centrosymmetric. N—H⋯O hydrogen bonds link the diimidazolium cations and the polyoxidoanions into a chain structure along [100]. π–π inter­actions between the imidazole rings and between the imidazole and benzene rings [centroid–centroid distances = 3.611 (2) and 3.689 (3) Å, respectively] connect the chains.

## Related literature

For general background to polyoxidometalate-based organic-inorganic hybrid compounds, see: Xie *et al.* (2011 ▶); Xu *et al.* (1999 ▶). For the synthesis of the ligand, see: Yang *et al.* (2006 ▶).
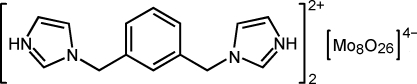


## Experimental

### 

#### Crystal data

(C_14_H_16_N_4_)_2_[Mo_8_O_26_]
*M* *_r_* = 1664.14
Monoclinic, 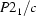

*a* = 12.163 (2) Å
*b* = 12.785 (3) Å
*c* = 14.937 (3) Å
β = 96.82 (3)°
*V* = 2306.3 (8) Å^3^
*Z* = 2
Mo *K*α radiation
μ = 2.20 mm^−1^
*T* = 293 K
0.12 × 0.10 × 0.10 mm

#### Data collection

Rigaku R-AXIS RAPID diffractometer
Absorption correction: multi-scan (*ABSCOR*; Higashi, 1995 ▶) *T*_min_ = 0.780, *T*_max_ = 0.809
21595 measured reflections
5261 independent reflections
4579 reflections with *I* > 2σ(*I*)
*R*_int_ = 0.032

#### Refinement

*R*[*F* ^2^ > 2σ(*F* ^2^)] = 0.026
*wR*(*F* ^2^) = 0.057
*S* = 1.01
5261 reflections
324 parameters
2 restraints
H atoms treated by a mixture of independent and constrained refinement
Δρ_max_ = 1.12 e Å^−3^
Δρ_min_ = −1.30 e Å^−3^

### 

Data collection: *RAPID-AUTO* (Rigaku, 1998 ▶); cell refinement: *RAPID-AUTO*; data reduction: *CrystalClear* (Rigaku/MSC, 2002 ▶); program(s) used to solve structure: *SHELXS97* (Sheldrick, 2008 ▶); program(s) used to refine structure: *SHELXL97* (Sheldrick, 2008 ▶); molecular graphics: *DIAMOND* (Brandenburg, 1999 ▶); software used to prepare material for publication: *SHELXTL* (Sheldrick, 2008 ▶).

## Supplementary Material

Crystal structure: contains datablock(s) I, global. DOI: 10.1107/S1600536811053050/hy2492sup1.cif

Structure factors: contains datablock(s) I. DOI: 10.1107/S1600536811053050/hy2492Isup2.hkl

Additional supplementary materials:  crystallographic information; 3D view; checkCIF report

## Figures and Tables

**Table 1:** Hydrogen-bond geometry (Å, °)

*D*—H⋯*A*	*D*—H	H⋯*A*	*D*⋯*A*	*D*—H⋯*A*
N2—H21⋯O12^i^	0.90 (1)	1.96 (2)	2.844 (4)	168 (4)
N4—H41⋯O5^ii^	0.90 (1)	2.51 (5)	3.003 (5)	115 (4)
N4—H41⋯O8^ii^	0.90 (1)	2.23 (4)	2.909 (5)	132 (5)

Symmetry codes: (i) 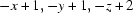; (ii) 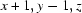.

**Fig. 1.**
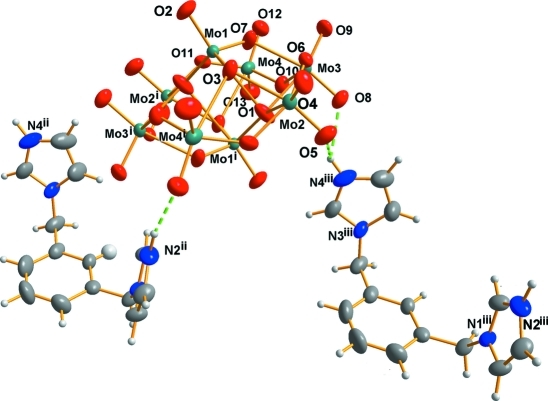
The molecular structure of the title compound, showing displacement ellipsoids at the 50% probability level. Dashed lines denote hydrogen bonds. [Symmetry codes: (i) 1-x, 2-y, 2-z; (ii) x, 1+y, z; (iii) -1+x, 1+y, z.]

**Fig. 2.**
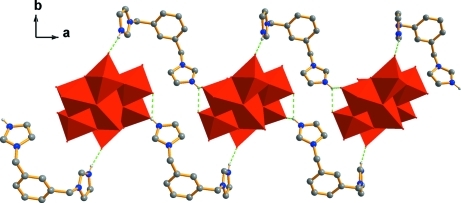
A view of the hydrogen-bonded chain structure along [1 0 0]. Dashed lines denote hydrogen bonds.

## supplementary crystallographic information

### Comment

The synthesis and characterization of coordination networks based on the idea of self-assembly of specifically designed building blocks have been an area of rapid growth in recent years. In the last decades, more and more attention has been paid to the rational design and assembly of new polyoxometalate(POM)-based organic-inorganic hybrid compounds due to their structural diversities and abundant potential applications in catalysis, ion exchange, sorption and magnetism (Xie *et al.*, 2011). Octamolybdate family with a variety of structural isomers is a kind of important POMs building blocks (Xu *et al.*, 1999). The title compound was synthesized at a low pH value condition, as an unexpected product during the process of preparing POM-based Cu(II)–ligand complex. We report its structure here.

The asymmertric unit of the title compound contains one half of β-[Mo_8_O_26_]^4-^ polyoxoanion and one (1,3-phenylenedimethylene)-di-1*H*-imidazolium cation (Fig. 1). The polyoxoanion is centrosymmetric. N—H···O hydrogen bonds link the cations and polyoxoanions into a chain structure along [1 0 0] (Fig. 2, Table 1). π–π interactions between the imidazole rings and between the imidazole and benzene rings [centroid–centroid distances = 3.611 (2) and 3.689 (3) Å] connect the chains.

### Experimental

The 1,3-bis(imidazol-l-yl-methyl)benzene (bimb) ligand was synthesized following the literature method (Yang *et al.*, 2006). The title compound was synthesized by mixing bimb (0.101 g, 0.5 mmol), Cu(NO_3_)_2_.4H_2_O (0.102 g, 0.05 mmol), sodium molybdate (0.505 g, 2.5 mmol), H_2_O (8 ml) and ethanol (2 ml) and stirring at room temperature for 10 min. The pH value of the mixture was adjusted to 2.0 with 1M HCl, and then the mixture was sealed in a Teflon-lined autoclave and heated at 125°C for 4 days. After slow cooling to room temperature, black block crystals were obtained in 22% yield based on Mo atoms.

### Refinement

The electron density residual peak (1.12) and hole (-1.30) are all around of Mo4 atom with distances of 0.71 and 0.81 Å, respectively. H atoms bound to C atoms were placed in calculated positions and treated as riding on their parent atoms, with C—H = 0.93 (aromatic) and 0.97 (methylene) Å and with *U*_iso_(H) = 1.2*U*_eq_(C). H atoms bound to N atoms were located from a difference Fourier map and refined isotropically.

### Crystal data

**Table:**

(C_14_H_16_N_4_)_2_[Mo_8_O_26_]	*F*(000) = 1600
*M**_r_* = 1664.14	*D*_x_ = 2.396 Mg m^−^^3^
Monoclinic, *P*2_1_/*c*	Mo *K*α radiation, λ = 0.71073 Å
Hall symbol: -P 2ybc	Cell parameters from 17887 reflections
*a* = 12.163 (2) Å	θ = 3.1–27.5°
*b* = 12.785 (3) Å	µ = 2.20 mm^−^^1^
*c* = 14.937 (3) Å	*T* = 293 K
β = 96.82 (3)°	Block, colorless
*V* = 2306.3 (8) Å^3^	0.12 × 0.10 × 0.10 mm
*Z* = 2	

### Data collection

**Table:**

Rigaku R-AXIS RAPID diffractometer	5261 independent reflections
Radiation source: fine-focus sealed tube	4579 reflections with *I* > 2σ(*I*)
graphite	*R*_int_ = 0.032
ω scan	θ_max_ = 27.5°, θ_min_ = 3.1°
Absorption correction: multi-scan (*ABSCOR*; Higashi, 1995)	*h* = −15→15
*T*_min_ = 0.780, *T*_max_ = 0.809	*k* = −16→15
21595 measured reflections	*l* = −19→19

### Refinement

**Table:**

Refinement on *F*^2^	Primary atom site location: structure-invariant direct methods
Least-squares matrix: full	Secondary atom site location: difference Fourier map
*R*[*F*^2^ > 2σ(*F*^2^)] = 0.026	Hydrogen site location: inferred from neighbouring sites
*wR*(*F*^2^) = 0.057	H atoms treated by a mixture of independent and constrained refinement
*S* = 1.01	*w* = 1/[σ^2^(*F*_o_^2^) + (0.0219*P*)^2^ + 3.6973*P*] where *P* = (*F*_o_^2^ + 2*F*_c_^2^)/3
5261 reflections	(Δ/σ)_max_ = 0.003
324 parameters	Δρ_max_ = 1.12 e Å^−^^3^
2 restraints	Δρ_min_ = −1.30 e Å^−^^3^

### Fractional atomic coordinates and isotropic or equivalent isotropic displacement parameters (Å^2^)

**Table:**

	*x*	*y*	*z*	*U*_iso_*/*U*_eq_	
C1	0.6205 (3)	0.5883 (3)	1.0719 (2)	0.0442 (9)	
H1	0.6032	0.6216	1.1237	0.053*	
C2	0.6426 (3)	0.6343 (3)	0.9957 (2)	0.0411 (8)	
H2	0.6443	0.7059	0.9850	0.049*	
C3	0.6529 (3)	0.4656 (3)	0.9766 (3)	0.0414 (8)	
H3	0.6623	0.4003	0.9512	0.050*	
C4	0.6943 (3)	0.5749 (3)	0.8462 (2)	0.0375 (8)	
H4A	0.6747	0.6456	0.8269	0.045*	
H4B	0.6542	0.5268	0.8039	0.045*	
C5	0.8175 (3)	0.5588 (3)	0.8459 (2)	0.0334 (7)	
C6	0.8932 (4)	0.6252 (3)	0.8933 (3)	0.0574 (11)	
H6	0.8687	0.6820	0.9245	0.069*	
C7	1.0057 (4)	0.6069 (4)	0.8944 (4)	0.0722 (15)	
H7	1.0564	0.6506	0.9275	0.087*	
C8	1.0431 (3)	0.5248 (4)	0.8469 (4)	0.0638 (13)	
H8	1.1188	0.5140	0.8473	0.077*	
C9	0.9687 (3)	0.4581 (3)	0.7984 (3)	0.0416 (9)	
C10	0.8560 (3)	0.4767 (3)	0.7982 (2)	0.0332 (7)	
H10	0.8054	0.4328	0.7652	0.040*	
C11	1.0073 (3)	0.3659 (3)	0.7484 (3)	0.0517 (11)	
H11A	0.9529	0.3499	0.6974	0.062*	
H11B	1.0764	0.3833	0.7254	0.062*	
C12	0.9427 (4)	0.2172 (4)	0.8436 (4)	0.0673 (14)	
H12	0.8670	0.2300	0.8342	0.081*	
C13	0.9922 (5)	0.1420 (4)	0.8937 (4)	0.0722 (15)	
H13	0.9581	0.0920	0.9264	0.087*	
C14	1.1198 (3)	0.2305 (3)	0.8354 (3)	0.0503 (10)	
H14	1.1882	0.2521	0.8202	0.060*	
Mo1	0.547848 (19)	0.90288 (2)	1.084672 (17)	0.02207 (6)	
Mo2	0.30667 (2)	1.04699 (2)	1.134611 (18)	0.02643 (7)	
Mo3	0.27124 (2)	0.86907 (2)	0.973110 (19)	0.02576 (7)	
Mo4	0.45286 (2)	0.80953 (3)	0.81444 (2)	0.03626 (8)	
N1	0.6622 (2)	0.5573 (2)	0.93667 (18)	0.0312 (6)	
N2	0.6281 (3)	0.4835 (3)	1.0589 (2)	0.0463 (8)	
N3	1.0240 (2)	0.2736 (3)	0.8073 (2)	0.0425 (7)	
N4	1.1028 (4)	0.1517 (3)	0.8884 (3)	0.0622 (10)	
O1	0.6277 (2)	0.9749 (2)	1.01029 (18)	0.0472 (7)	
O2	0.6373 (2)	0.8237 (2)	1.14858 (18)	0.0477 (7)	
O3	0.48333 (18)	0.9874 (2)	1.15775 (16)	0.0372 (6)	
O4	0.2810 (2)	1.0400 (2)	1.24323 (16)	0.0439 (6)	
O5	0.1925 (2)	1.1056 (2)	1.08042 (19)	0.0500 (7)	
O6	0.27884 (17)	0.90605 (17)	1.09648 (15)	0.0298 (5)	
O7	0.45232 (19)	0.82404 (19)	1.01803 (19)	0.0429 (6)	
O8	0.1530 (2)	0.9344 (2)	0.93423 (17)	0.0417 (6)	
O9	0.2328 (2)	0.7420 (2)	0.97568 (19)	0.0439 (6)	
O10	0.33456 (19)	0.8728 (2)	0.85883 (16)	0.0361 (5)	
O11	0.6000 (2)	0.82712 (18)	0.86168 (17)	0.0371 (5)	
O12	0.4326 (2)	0.6786 (2)	0.8263 (2)	0.0548 (7)	
O13	0.4385 (3)	0.8367 (2)	0.70317 (19)	0.0555 (7)	
H21	0.619 (4)	0.433 (3)	1.099 (2)	0.066 (14)*	
H41	1.155 (3)	0.107 (4)	0.911 (4)	0.10 (2)*	

### Atomic displacement parameters (Å^2^)

**Table:**

	*U*^11^	*U*^22^	*U*^33^	*U*^12^	*U*^13^	*U*^23^
C1	0.052 (2)	0.054 (2)	0.0275 (18)	0.0126 (18)	0.0089 (15)	0.0002 (17)
C2	0.058 (2)	0.0311 (18)	0.035 (2)	0.0116 (16)	0.0088 (16)	−0.0002 (15)
C3	0.051 (2)	0.0339 (19)	0.039 (2)	0.0056 (16)	0.0056 (16)	0.0060 (15)
C4	0.0397 (19)	0.046 (2)	0.0268 (17)	0.0124 (15)	0.0046 (14)	0.0046 (15)
C5	0.0370 (18)	0.0339 (18)	0.0295 (17)	0.0007 (14)	0.0049 (13)	0.0056 (14)
C6	0.062 (3)	0.047 (2)	0.064 (3)	−0.013 (2)	0.012 (2)	−0.013 (2)
C7	0.048 (3)	0.074 (3)	0.093 (4)	−0.028 (2)	0.001 (2)	−0.012 (3)
C8	0.031 (2)	0.082 (4)	0.079 (3)	−0.004 (2)	0.010 (2)	0.010 (3)
C9	0.0353 (19)	0.052 (2)	0.039 (2)	0.0081 (16)	0.0115 (15)	0.0141 (17)
C10	0.0288 (16)	0.043 (2)	0.0277 (17)	0.0024 (14)	0.0033 (12)	0.0024 (14)
C11	0.049 (2)	0.063 (3)	0.047 (2)	0.022 (2)	0.0226 (18)	0.016 (2)
C12	0.044 (2)	0.076 (3)	0.087 (4)	0.012 (2)	0.025 (2)	0.026 (3)
C13	0.085 (4)	0.059 (3)	0.081 (4)	0.021 (3)	0.044 (3)	0.024 (3)
C14	0.038 (2)	0.056 (3)	0.057 (3)	0.0151 (18)	0.0060 (18)	0.006 (2)
Mo1	0.02070 (12)	0.02499 (13)	0.02034 (12)	0.00065 (9)	0.00169 (9)	0.00309 (10)
Mo2	0.02634 (13)	0.03154 (14)	0.02250 (13)	0.00129 (10)	0.00740 (10)	−0.00118 (11)
Mo3	0.02085 (12)	0.02662 (13)	0.03005 (15)	−0.00019 (10)	0.00399 (10)	−0.00093 (11)
Mo4	0.03172 (15)	0.04101 (17)	0.03793 (17)	0.01049 (12)	0.01196 (12)	0.01119 (13)
N1	0.0332 (14)	0.0329 (15)	0.0278 (14)	0.0086 (11)	0.0043 (11)	0.0027 (11)
N2	0.0480 (19)	0.052 (2)	0.0386 (18)	−0.0027 (15)	0.0048 (14)	0.0181 (15)
N3	0.0321 (15)	0.0544 (19)	0.0423 (18)	0.0143 (14)	0.0101 (13)	0.0102 (15)
N4	0.072 (3)	0.057 (2)	0.057 (2)	0.030 (2)	0.005 (2)	0.0138 (19)
O1	0.0685 (18)	0.0394 (14)	0.0395 (15)	−0.0240 (13)	0.0304 (13)	−0.0137 (11)
O2	0.0417 (14)	0.0540 (17)	0.0440 (16)	0.0217 (12)	−0.0092 (11)	0.0005 (13)
O3	0.0266 (12)	0.0462 (14)	0.0375 (14)	0.0049 (10)	−0.0015 (9)	−0.0123 (11)
O4	0.0429 (14)	0.0641 (18)	0.0268 (13)	−0.0067 (12)	0.0124 (10)	−0.0035 (12)
O5	0.0521 (16)	0.0485 (16)	0.0464 (16)	0.0204 (13)	−0.0062 (12)	−0.0066 (13)
O6	0.0279 (11)	0.0316 (12)	0.0299 (12)	−0.0047 (9)	0.0038 (9)	0.0041 (9)
O7	0.0268 (12)	0.0363 (14)	0.0635 (18)	0.0056 (10)	−0.0038 (11)	−0.0175 (12)
O8	0.0347 (13)	0.0524 (16)	0.0370 (14)	0.0147 (11)	−0.0001 (10)	−0.0028 (12)
O9	0.0382 (13)	0.0332 (13)	0.0588 (17)	−0.0100 (10)	−0.0004 (12)	−0.0036 (12)
O10	0.0316 (12)	0.0479 (15)	0.0290 (12)	0.0017 (10)	0.0045 (9)	−0.0064 (11)
O11	0.0449 (14)	0.0251 (11)	0.0435 (15)	0.0036 (10)	0.0149 (11)	−0.0008 (10)
O12	0.0512 (16)	0.0449 (16)	0.070 (2)	−0.0018 (13)	0.0158 (14)	0.0089 (14)
O13	0.073 (2)	0.0581 (18)	0.0390 (16)	0.0120 (15)	0.0235 (14)	0.0048 (14)

### Geometric parameters (Å, °)

**Table:**

C1—C2	1.336 (5)	C13—H13	0.9300
C1—N2	1.359 (5)	C14—N3	1.312 (5)
C1—H1	0.9300	C14—N4	1.313 (6)
C2—N1	1.362 (4)	C14—H14	0.9300
C2—H2	0.9300	Mo1—O2	1.695 (2)
C3—N2	1.319 (5)	Mo1—O7	1.756 (2)
C3—N1	1.326 (4)	Mo1—O3	1.783 (2)
C3—H3	0.9300	Mo1—O1	1.811 (2)
C4—N1	1.468 (4)	Mo2—O4	1.690 (2)
C4—C5	1.513 (5)	Mo2—O5	1.697 (3)
C4—H4A	0.9700	Mo2—O6	1.908 (2)
C4—H4B	0.9700	Mo2—O11^i^	1.966 (2)
C5—C10	1.381 (5)	Mo2—O3	2.267 (2)
C5—C6	1.384 (5)	Mo2—O1^i^	2.410 (3)
C6—C7	1.387 (7)	Mo3—O9	1.692 (2)
C6—H6	0.9300	Mo3—O8	1.704 (2)
C7—C8	1.374 (7)	Mo3—O6	1.894 (2)
C7—H7	0.9300	Mo3—O10	1.955 (2)
C8—C9	1.383 (6)	Mo3—O7	2.298 (2)
C8—H8	0.9300	Mo3—O1^i^	2.341 (2)
C9—C10	1.391 (5)	Mo4—O13	1.687 (3)
C9—C11	1.501 (6)	Mo4—O12	1.704 (3)
C10—H10	0.9300	Mo4—O10	1.842 (2)
C11—N3	1.472 (5)	Mo4—O11	1.858 (3)
C11—H11A	0.9700	N2—H21	0.901 (10)
C11—H11B	0.9700	N4—H41	0.896 (10)
C12—C13	1.320 (7)	O1—Mo3^i^	2.341 (2)
C12—N3	1.385 (5)	O1—Mo2^i^	2.410 (2)
C12—H12	0.9300	O11—Mo2^i^	1.966 (2)
C13—N4	1.364 (7)		
			
C2—C1—N2	106.7 (3)	O4—Mo2—O6	100.96 (12)
C2—C1—H1	126.6	O5—Mo2—O6	99.70 (12)
N2—C1—H1	126.6	O4—Mo2—O11^i^	100.83 (12)
C1—C2—N1	107.6 (3)	O5—Mo2—O11^i^	95.11 (13)
C1—C2—H2	126.2	O6—Mo2—O11^i^	149.63 (10)
N1—C2—H2	126.2	O4—Mo2—O3	96.62 (11)
N2—C3—N1	107.9 (3)	O5—Mo2—O3	158.19 (12)
N2—C3—H3	126.1	O6—Mo2—O3	81.93 (9)
N1—C3—H3	126.1	O11^i^—Mo2—O3	74.76 (10)
N1—C4—C5	110.7 (3)	O4—Mo2—O1^i^	166.93 (12)
N1—C4—H4A	109.5	O5—Mo2—O1^i^	87.85 (12)
C5—C4—H4A	109.5	O6—Mo2—O1^i^	71.84 (9)
N1—C4—H4B	109.5	O11^i^—Mo2—O1^i^	82.45 (9)
C5—C4—H4B	109.5	O3—Mo2—O1^i^	71.88 (10)
H4A—C4—H4B	108.1	O9—Mo3—O8	104.73 (13)
C10—C5—C6	119.0 (4)	O9—Mo3—O6	101.50 (12)
C10—C5—C4	120.1 (3)	O8—Mo3—O6	98.69 (11)
C6—C5—C4	121.0 (3)	O9—Mo3—O10	100.47 (12)
C5—C6—C7	119.9 (4)	O8—Mo3—O10	95.41 (11)
C5—C6—H6	120.1	O6—Mo3—O10	149.84 (10)
C7—C6—H6	120.1	O9—Mo3—O7	90.60 (11)
C8—C7—C6	120.6 (4)	O8—Mo3—O7	164.34 (12)
C8—C7—H7	119.7	O6—Mo3—O7	81.03 (10)
C6—C7—H7	119.7	O10—Mo3—O7	78.34 (10)
C7—C8—C9	120.3 (4)	O9—Mo3—O1^i^	163.48 (12)
C7—C8—H8	119.8	O8—Mo3—O1^i^	91.69 (12)
C9—C8—H8	119.8	O6—Mo3—O1^i^	73.73 (9)
C8—C9—C10	118.6 (4)	O10—Mo3—O1^i^	79.36 (10)
C8—C9—C11	121.4 (4)	O7—Mo3—O1^i^	73.12 (10)
C10—C9—C11	120.0 (4)	O13—Mo4—O12	107.75 (16)
C5—C10—C9	121.5 (3)	O13—Mo4—O10	105.68 (13)
C5—C10—H10	119.2	O12—Mo4—O10	105.41 (13)
C9—C10—H10	119.2	O13—Mo4—O11	109.38 (14)
N3—C11—C9	111.1 (3)	O12—Mo4—O11	102.95 (12)
N3—C11—H11A	109.4	O10—Mo4—O11	124.66 (10)
C9—C11—H11A	109.4	C3—N1—C2	108.4 (3)
N3—C11—H11B	109.4	C3—N1—C4	126.6 (3)
C9—C11—H11B	109.4	C2—N1—C4	124.9 (3)
H11A—C11—H11B	108.0	C3—N2—C1	109.3 (3)
C13—C12—N3	107.7 (4)	C3—N2—H21	124 (3)
C13—C12—H12	126.2	C1—N2—H21	127 (3)
N3—C12—H12	126.2	C14—N3—C12	107.7 (4)
C12—C13—N4	106.7 (4)	C14—N3—C11	125.6 (3)
C12—C13—H13	126.7	C12—N3—C11	126.7 (3)
N4—C13—H13	126.7	C14—N4—C13	109.4 (4)
N3—C14—N4	108.6 (4)	C14—N4—H41	125 (4)
N3—C14—H14	125.7	C13—N4—H41	125 (4)
N4—C14—H14	125.7	Mo1—O1—Mo3^i^	132.92 (13)
O2—Mo1—O7	108.29 (13)	Mo1—O1—Mo2^i^	138.66 (13)
O2—Mo1—O3	108.47 (12)	Mo3^i^—O1—Mo2^i^	88.18 (8)
O7—Mo1—O3	112.67 (11)	Mo1—O3—Mo2	126.04 (12)
O2—Mo1—O1	107.09 (14)	Mo3—O6—Mo2	120.84 (11)
O7—Mo1—O1	108.11 (13)	Mo1—O7—Mo3	124.65 (12)
O3—Mo1—O1	111.99 (12)	Mo4—O10—Mo3	135.18 (14)
O4—Mo2—O5	104.33 (14)	Mo4—O11—Mo2^i^	129.54 (13)

Symmetry codes: (i) −*x*+1, −*y*+2, −*z*+2.

### Hydrogen-bond geometry (Å, °)

**Table:**

*D*—H···*A*	*D*—H	H···*A*	*D*···*A*	*D*—H···*A*
N2—H21···O12^ii^	0.90 (1)	1.96 (2)	2.844 (4)	168 (4)
N4—H41···O5^iii^	0.90 (1)	2.51 (5)	3.003 (5)	115 (4)
N4—H41···O8^iii^	0.90 (1)	2.23 (4)	2.909 (5)	132 (5)

Symmetry codes: (ii) −*x*+1, −*y*+1, −*z*+2; (iii) *x*+1, *y*−1, *z*.
